# Teamwork and Safety Climate in Homecare: A Mixed Method Study

**DOI:** 10.3390/ijerph15112495

**Published:** 2018-11-08

**Authors:** Agneta Larsson, Mats Westerberg, Lena Karlqvist, Gunvor Gard

**Affiliations:** 1 Division of Health and Rehabilitation, Department of Health Sciences, Luleå University of Technology, 97187 Luleå, Sweden; lena.karlqvist@hotmail.com (L.K.); gunvor.gard@ltu.se (G.G.); 2 Division of Innovation and Design, Department of Business Administration, Technology and Social Sciences, Luleå University of Technology, 97187 Luleå, Sweden; mats.westerberg@ltu.se

**Keywords:** health services research, risk management, safety climate, teamwork, quality improvement, mental strain, injury

## Abstract

A rapidly changing homecare service sector implies difficulties to control safety and health risks for staff and to guarantee standardised deliveries of services to recipients. This study aimed to describe staff perceptions of safety climate and practices in homecare service teams, and suggestions for improvements. A second aim was to identify if and how the appraisals of safety climate were related to individual perceptions of safety, mental strain and adverse events/injury. A convergent parallel mixed methods design was used. Nursing assistants and care aides (133 in total, representing 11 work teams) in the north of Sweden replied to a survey and participated in focus group interviews. Results were analysed with ANOVA (inter-team differences) and by qualitative content analysis. Significant diversity was identified between the teams in five of seven dimensions of safety climate. Important areas for improvement were: a need to define and agree on criteria for a safe working environment; leadership prioritising safety at work; and management able to provide trust, support and time. A prerequisite for these agreements was improved authority and communication between all parties involved. The safety climate dimensions were related to personal perceptions of safety and mental strain and, partly, to adverse events/injuries.

## 1. Introduction

The homecare service sector is rapidly changing in several Western societies. Economic and demographic changes and advances in medical technology have led to an allocation of social and medical care in people´s homes [1,2]. Regulation and policy differ between countries and according to the variety of the service provided. In Sweden, the municipalities are responsible for domestic and personal care, and the County Councils for home health care [1]. The great variety of home environments implies difficulties for the homecare staff to manage safety and health risks and to guarantee standardised deliveries of services of equal quality [2].

In jobs with unpredicted environmental challenges and care recipients with a variety of needs, not all home care and rehabilitation processes can be standardised. Thus, a range of possible alternatives for how to perform the services, as well as strong self-efficacy beliefs for safe work practices among staff, is crucial for high service quality and safety [3,4]. The opportunities for the staff to control job content and job conditions have been shown to influence their work practices and health [5]. From this view, it is important to consider the actual opportunities for control that are allowed within the organisation [4]. The safety climate is one aspect of the organisational climate. It is proposed that shared climate perceptions emerge from social interaction among members of a social unit, such as a work team, trying to make sense of complex work situations. It is created by shared perceptions about what procedures, practices, and behaviours get rewarded and supported in the organisation [6,7]. As such, a safety climate can be defined as the shared perceptions of safety-related policies and practices influencing safety in the organisation [7]. It is often classified in different dimensions, and includes, for example, staff safety priorities and refusal to accept risks, and the ability of management to prioritise safety over productivity [8].

Previous research shows that shared perceptions of the management-related safety climate has a significant influence on safety climate dimensions at the workgroup level, and hence indirectly affect the safety behaviour of individual workers [9,10,11]. In groups with a more advanced safety climate, it is plausible also to expect better safety-related practices and thus a lower injury rate [12]. Besides a shared climate, other factors such as high levels of perceived stress at work are reported to be of significance for the risk of injuries being inflicted [13]. In the homecare sector, occupational injuries and hazards are significant problems. Staff report musculoskeletal strain from client-care tasks, slips, trips and falls, car accidents, exposure to chemical or infectious agents or violence [14,15,16].

So far, research on safety culture/climate has mostly been conducted in institutional settings such as hospitals, which has resulted in a significant knowledge gap about safety in homecare. Hospital safety climate studies are becoming a key factor in the improvement and maintenance of staff’s safety perceptions within units, as well as within hospitals [17]. Safety climate may vary markedly across units and clinical specialisms within individual hospitals, as well as between hospitals [17,18,19,20]. Further, there is emerging evidence that interventions which focus on modifiable aspects of the safety climate can increase the health and safety of medical care staff, as well as of their patients [21,22,23]. The organisational processes to improve hospital staff occupational safety and patient safety are recommended to be performed in parallel [14,24].

Increased knowledge about the safety climate in municipality-run homecare services for the elderly can enhance the safety and quality of the working practices in this sector.

The first aim of this study is to describe homecare staff perceptions of safety climate and practices in homecare service teams, and to make suggestions for improvements. A second aim is to identify if and how the perceptions of safety climate are related to individual perceptions of safety, mental strain and adverse events/injuries.

## 2. Materials and Methods

### 2.1. Population, Procedure and Ethics

A convergent parallel mixed methods design [25] was used to answer the first aim, by combining data from a survey and focus group interviews. This design was chosen as the combination of quantitative and qualitative data provides a deeper understanding of the topic. The second aim was answered by a quantitative analysis of survey data.

This research was conducted within a larger project for health and safety promotion in municipality-run homecare services for the elderly in the north of Sweden [26]. In total, 350 nursing aides and assistant nurses provided personal care and domestic aid to about 900 elderly people. The staff was divided into 18 teams and night patrols that were situated in the centre of the town or the countryside and was managed by 16 managers and one head of homecare services.

Of the total population, 298 were invited to answer the survey as they met the criterion of having worked in the same team for the previous six months. A letter containing information, a consent form, a hard copy of the survey and a prepaid envelope was provided via their supervisors in February 2009. The response rate was 53% (158) and varied between different teams. Of these, only the 11 teams with an acceptable response rate (more than six replies) were included, implying that 133 (45%) of the participants were included in this study. Six months later, each of the 11 teams was asked, through their manager, to choose two group members to represent their team in focus group discussions. They were provided with information about the time and topics of the focus groups. The research was approved by the Committee of Research Ethics at Umeå University, Sweden (Dnr 08-217 Ö).

### 2.2. Data Collection and Analysis

#### 2.2.1. Survey

Data were obtained through a comprehensive self-administrated questionnaire covering individual background factors, job-related factors, individual resources and health-related factors [26]. The items and scales analysed in the present paper are described below.

*The shared perceptions of the safety climate* were measured using the 50 items of the Nordic Safety Climate Questionnaire (NOSACQ-50) [8] graded on four-point scales (end points: 1 = ‘fully disagree’ and 4 = ‘fully agree’). The respondent was considered to be a representative reporter of his or her social unit’s (homecare team’s) shared perceptions of the safety climate. Together, these items produced measures of seven dimensions of the safety climate, of which three represented the management level and four the team levels [8]. As a result of a face validity test, the text ‘the efficiency in medical care and services work’ was added within brackets after one item to elucidate ‘production’ in this context. Analyses of the internal consistency reliability estimate of the scale resulted in Cronbach’s alpha values above α 0.7 for all seven dimensions, which was considered acceptable.

The mean, standard deviation and frequency measures were used to analyse the data. The levels of safety climate were defined by using the limit values proposed by the NOSACQ guidelines [27]. To obtain a representative value of the teams’ shared perceptions of safety climate, we excluded seven teams in which fewer than six respondents replied. In the remaining 11 teams, the response rate was ≥47%.

Between-team differences in terms of mean values for each of the seven safety climate dimensions were analysed by ANOVA, performed on the 11 teams.

Five-point scales were used to estimate current perceptions of mental strain and general safety. Personal *work-related mental strain* was measured by the single-item question: ‘Stress means a situation in which a person feels tense, restless, nervous or anxious or is unable to sleep at night because his/her mind is troubled all the time. Do you feel this kind of stress these days *because of your work*?’ (end points: 1 = ‘not at all’ and 5 = ‘a lot’) [28]. The general level of *safety at work* was measured by a single-item question: ‘Provide a general assessment of the safety of your workplace.‘ (end points: 1 = ‘very bad’ and 5 = ‘excellent’) derived from Olsen [23]. *Injury event* in the last six months was measured by two items (‘Have you been involved in any accident or incident during the last six months? ‘, ‘Did you get hurt due to the event/events?”) in which the respondents answered ‘yes’ or ‘no’ and, if relevant, described the adverse event/events. A third item assessed outcome regarding the loss of time from work. ‘If you were injured, did you have to cancel your work for at least an hour?‘ (no/yes but not reporting sick/reported sick three days or less/more than three days) [29]. The answers were classified into six categories to produce a six-point variable ranging from zero (no injury) to five (injury time loss > 3 days).

The variables safety at work, mental strain and injury event were prioritised as the main health and safety outcome measures at employee level according to earlier research.

Relationships between variables were explored with Pearson’s correlation coefficient (2-tailed). The software SPSS version 17.0 was used, with a statistical significance of *p* < 0.05.

#### 2.2.2. Focus Groups

In all, four focus group sessions were conducted in a meeting room at the social administration’s premises. Initially, the confidentiality of group discussions was discussed, and the participants assured that no opinions of individual participants would be shared outside the session (e.g., with managers). However, the results were aimed to be reported and used for further discussions and quality development work within the organisation. Two major topics were presented to the discussants, one at a time, by the facilitator; (1) to describe how they perceived and managed their work situation and (2) to identify positive factors and activities that could be further developed to promote the front-line staff’s health and safety at work. The questions were open-ended and were intended to allow for a free exchange of ideas [30]. Each focus group session lasted two hours and was audiotaped and transcribed. The data analysis was made by manifest qualitative content analysis. This is a process that can be described as identifying, coding and categorising the primary pattern in the data, i.e., the content [31].

## 3. Results

### 3.1. Study Participants

The survey respondents had a mean age of 45 (±10.8, range 21–67) years, the majority (92%) were women, 57% were assistant nurses and 43% were nursing aides. Their seniority in their present team was a mean 8.8 (±7.5, range 0.5–33) years, and the teams consisted of a mean 26.5 (±11.1, range 14–46) co-workers.

The focus group participants were nursing aides and assistant nurses with a mean age of 46 years and the majority were women (one man), representing 11 teams.

### 3.2. Survey Data

The shared perceptions of the safety climate and inter-unit differences.

In the whole sample, mean values above 3.0 were found for all dimensions of the safety climate. This indicates a generally ‘fairly good’ climate according to reference values. However, statistically significant inter-unit differences were exhibited for five dimensions, where the highest and lowest mean values varied between 2.6 and 3.8. The highest mean values across the whole sample, and also at the unit level (all group means were above 3.1), were in ‘staff confidence in peers to communicate and learn from experiences‘ and ‘trust in each other’s ability to ensure safety in everyday work‘ (dimension 6), and ‘belief in that formal safety systems—e.g., safety rounds—are effective‘ (dimension 7). They also exhibited confidence in their managers’ desire to treat employees fairly when reporting an accident (dimension 3). The dimensions with the lowest mean values across the whole sample (3.0–3.1) were ‘staff opinion of management’s priorities and attainments concerning safety‘ (dimension 1) and of ‘management’s provision of encouragement and support for staff participation in safety management‘ (dimension 2). The same applied to ‘staff’s safety priorities and refusal to accept risks‘ (dimension 5) (Table 1).

Post hoc analyses (Tukey HSD, significance level 0.05) showed that, in the main, five teams accounted for the significant inter-unit differences. In the three management-level dimensions, team #11 (accompanied by team #3 in dimension 1) had lower appraisals of the climate than the other groups. Team #5 (accompanied by team #7 & #9 in dimensions 2 and 3) had the highest appraisals in comparison to the other groups. In the two team-level dimensions of worker safety commitment and priority, respectively, team #3 stood out as low, and again, team #5 as high. The main characteristics differentiating these ‘low’ and ‘high’ teams were: number of team members (highest: 46 in team #11, and lowest: 14 in team #5) and geographical location (teams #3 and #11 located in the city centre, and team #5 in the countryside).

The table shows respondents’ appraisals of their work unit’s shared perceptions of seven aspects of the safety climate’. The data are presented regarding the overall climate in the organisation and at the level of the homecare teams. The differences between 11 teams are illustrated with the highest and lowest team means, and with p-values^1^ reflecting the overall differences.

### 3.3. Focus Groups

#### 3.3.1. A Need to Define and Agree on Criteria for a Safe Working Environment

Diverse expectations concerning the job content were the cause of many dilemmas and conflicts. The staff recognised the importance of setting standards for safe work with more specific job descriptions. Also, they expected that all professionals involved, i.e., the social services administrator, managers and staff, needed to deliver the same message about the timing and content of service to each recipient and their respective relatives. Agreed, clear and reasonable requirements were considered of importance to be able to meet the expectations. Consequently, by fulfilling expectations they perceived job satisfaction and were equally and respectfully treated. Necessary workplace adjustments had to be specified, arranged and solved in advance, and the staff had to be well informed before beginning their work, and then continuously: ‘*This are the preconditions for us to have this (workplace adjustments arranged) at our workplace. It gives some power to be able to say that in advance.*’ A good functioning homecare work environment was experienced as a priority for the staffs’ safety and the recipient’s best interest. It had been noted that some recipients expressed concern when they saw that the staff were struggling. ‘*From an ethical view, it is important that the care recipient returns home to an environment where the homecare staff can help him or her in a good way. The most important thing is to take good care of the recipient. The staffs´ well-being is also important for the recipient to feel well*’.

#### 3.3.2. A Need for Leadership with Clear Safety Priorities

The respondents wanted their managers to act tougher in negotiations about how decided actions could be completed rapidly; for example, with the medical care about the handover and timing of when a service recipient was returning home from the hospital, as well as with the recipient/relatives about appropriate furnishing and cleaning equipment, or with the home care services administrator about the content the of expected services: ‘*We do a lot of things that we shouldn’t be doing, but we do it to get it done, we suffer and struggle quietly with narrow bathrooms and low beds, we do a really good job. We struggle against the relatives of the care recipient; we struggle against the recipient, we struggle against the occupational therapist, against the home care service administrator*’. The managers rarely raised issues about strategies for safe working practices in unexpected situations. Staff tried to remind peers to consider own health and safety in critical situations such as time pressure; e.g., not driving too fast, but say this should be the managers’ responsibility: ‘*… and then it is actually nonchalance by a manager if not caring for the staff*’. Instead, discrepancies in the delivery of service should be registered more frequently, and preconditions for safe practices improved.

#### 3.3.3. Need for Management Able to Provide Trust, Support and Time

In some teams, the staff were very pleased with the leadership and work practices that prioritised staff opportunities for inter-peer problem-solving. The preconditions for safe work could be improved by planning for a less tight schedule and time for scheduled and informal meetings: ‘*…our manager has scheduled 15–30 minutes after lunch for us to have the time to sit and talk and solve problems. That is really good for the benefit of the recipient and us so that we can provide good services … and we don’t have to feel guilty about it, because that is our job*’.

Teams described problems concerning leadership and called for stronger support, trust and positive feedback from their managers. They wished that the manager could act to be more supportive of the staff’s opportunities for teamwork and trust their judgements and competences. Those acting as safety representatives or assigned the responsibility to plan the weekly work schedules for their team were experienced and proud of their skills in logistics: ‘*I’m feeling satisfied when having managed to schedule more time allocated for the service to the recipients and minimised the time spent on travelling*’. They requested their managers to show a greater belief than today in staff’s competence to plan for safe work and high-quality service: ‘*If the manager makes changes in the schedule aiming to achieve higher efficiency of the team, it results in more running in all directions around town, more stressful for us, chaotic*’.

#### 3.3.4. Having a Say and Stakeholder Communication Is a Prerequisite for Safety Agreement

Lack of inter-peer communication and agreement was a problem within some teams, while other teams had a good supportive practice: ‘*There is no teamwork within homecare services whatsoever, I was shocked… you never meet peers and discuss the recipient‘, ‘This functions well in our team, we have good communication and are fairly united, receive work equipment fast, and have sound and distinct managers*’. Many were afraid of conflicts, and setting limits concerning the content of service if peers did not do the same was perceived as difficult. Some were afraid to be viewed as the ‘bad staff’ by the recipient by refusing to perform strenuous, dangerous, or non-prescribed tasks if their ‘good’ peers were providing the service.

The respondents emphasised their implicit skill in helping the recipients every day, even on days when the elderly person’s energy was lacking. They perceived that their unique knowledge about the homecare environment and recipients’ functional capacities was not often valued, or asked for when it came to the planning of services: ‘*The competence of those with long experience within homecare services should be valued higher; all decisions are taken on your own during a workday, all professional roles that you have, the competence is enormous, wide-ranging … a quality measure …’*.

Stakeholders’ dissimilar judgements of needs caused risks. More time, and arenas to discuss mutual strategies with external professionals, were requested. Lack of information from the medical care staff about the recipients’ condition, as well as organisational changes towards larger teams, were perceived as threats to the quality of service. Staff not being part of decision-making regarding service allocation caused conflicts. They wished that the manager could provide help with conflict resolution and transfer information in a better way, i.e., listen more to the staff’s opinion. Sometimes the process to solve practical issues was more rapid if staff by-passed their manager and phoned the medical nurses or occupational therapists directly.

### 3.4. Relations between Safety Climate and Personal Perceptions of Safety, Mental Strain and Injury Rate

The general level of safety at work was reported to be ‘acceptable’, with a mean value of 3.2 (±0.6) and the perceived mental strain was ‘fairly low’ 2.3 (±1.2). A total of 22 work-related accidents or incidents had happened during the previous six months, and these were reported by 14% of the respondents. Half of the events had led to injuries, of which five had resulted in time off work. Two of these injuries occurred outdoors and were associated with a car-driving accident and with slipping and falling on ice and snow. The other three adverse events occurred indoors and were primarily related to slipping and overexertion when moving and handling service recipients.

Further analysis showed that the safety climate dimensions were related to individual employees’ ratings of general safety at their workplace and individual perceptions of mental strain. There were also indications that reports of adverse events related to personal injury were related to the team’s shared perceptions of safety commitment, safety priority and non-risk acceptance and with personal perceptions of work-related mental strain (Table 2).

## 4. Discussion

### 4.1. Perceptions of Safety Climate, Practices, and Suggestions for Improvements.

In general, the safety climate in the overall study group in the homecare organisation was reported to be at, or above, a mean score of 3.0. This indicates fairly good levels, with only a slight need for improvement or maintenance [27]. Across all 11 teams, high levels of confidence concerning inter-peer communication, learning and trust in the ability of the group to manage safety were expressed. The teams also shared the belief that formal safety systems were efficient. Also, they had a fairly high belief in their managers’ ability to treat employees fairly when reporting an accident, even though significant inter-unit differences were found. This is in line with earlier findings [9,23], where higher appraisals in these three dimensions were reported in the field of medical care compared to the petroleum industry, although the safety climate was generally regarded to be lower within medical care. Analogously, the homecare group reported higher values in these three dimensions in comparison to the grand mean of responses from workers in general [27].

It is notable that a significant diversity was identified between the 11 homecare teams in five dimensions of the safety climate. The variations across teams was in line with research on medical organisations reporting varying safety climate across departments and specialisms [17,18,19,20]. Homecare teams reporting very good levels, 3.3 and above, concerning safety-related policies and practices can be interpreted as being in the lead in maintenance and proactive development [27]. This is promising, particularly as the teams that reported a more advanced safety climate can be in a good position to propose good solutions and practices for daily work to the other teams. It is plausible that improved communication and cooperation between homecare teams might have a positive impact on safety. Earlier research within medical care shows that, within teams, cooperation and safety-related practices play an important role in the prevention of adverse events and promoting workers’ well-being [19,32]. Also, focusing on fairness, learning and feedback loops in the system has been successful in improving safety [33]. It has been suggested that these factors can be enhanced by transitions and teamwork across units, which in turn are influenced by management expectations, actions and support concerning safety [9].

Even though factors relating to teamwork: the inter-peer safety communication, learning and competence, was estimated as high across the 11 teams, there were indications of other areas in great need of improvement in some teams, with appraisals of 2.7 and below. These are: staff’s commitment to safety, the priority they attribute to safety and refusal to accept risks. The same applies to the priority attributed to safety by management and their competence in managing safety, including means of empowering employees and supporting their participation.

The understanding of these results was deepened by the focus group results. The staff described experiences of distrust, inattention and negative feedback from the managers and external actors regarding homecare workers’ skills, concerns and ideas for improvement. More management support for safe work practices and actions, arenas and time for communication within teams and with external professionals, as well as influence on, and participation in, decisions were requested. The manager’s influence on the team safety climate has been confirmed in earlier research. There is strong evidence for a higher accident rate in workgroups who share the belief that the manager’s commitment to safety is low [34]. Nevertheless, peers’ commitment to safety can play an intervening role in the relationship between the manager’s influence on safety and staff safety behaviours [35], which is of particular interest in lone jobs, such as home care.

The high risk-acceptance in some teams in the present study can be understood by use of theories clarifying how perceptions of importance and interest, attitudes and subjective norms, as well as task-specific control, predict individual motivation towards a specific behaviour [36,37,38]. The staff emphasised their high competence in solving practical situations for the benefit of the recipient, often making decisions on their own (alone or in pairs). Setting limits on how to perform strenuous or hazardous tasks was perceived as a dilemma, due to the lack of immediate support from managers and/or peers; alternatively, if their closest peers choose to ignore safety regulations’. Agreement about the job content in practice and team commitment for safe work practices is essential. It was also evident that it is essential to elucidate further what constitutes a homecare team in this context. Homecare with high quality and safety is dependent on efficient coordination and cooperation with a network of external actors from the medical sector and other divisions of the social care and services sector.

In the focus groups, attention was drawn to a well-functioning environment and the well-being of the staff being key factors for a high-quality and ethical homecare service. This is in line with results from focus groups with medical care staff, addressing caregivers’ psychological and social capacities as prerequisites for patient safety [39]. Interventions consisting of safety checklists and low-tech client-handling devices are recommended [15,40].

The homecare staff wanted their managers, holding higher positional power, to value to a higher extent their opinions, and to negotiate for their benefit with other professionals and the recipients. This can be interpreted as a view of their managers as gatekeepers [41]. Consistently, results from the survey and focus groups in this study show that increased participation and authority to influence the work content were requested. Research has shown that a leader with a transformational style is effective in buffering organisational demands for production, by discussing conflicting demands and priorities with members of the work unit [41,42]. A scientific model has been developed and evaluated, where the indirect effects on perceived injury risk of physical risk factors, manager–employee exchange and empowerment were studied. Work-role stressors and upward safety communications were two important mediating variables, and manager–employee exchange and empowerment demonstrated significant indirect effects on perceived injury risk. An asymmetrical dualistic process was shown regarding the effect physical risk factors have on perceived injury risk via depletion of both psychological (i.e., role stressors) and physical resources (i.e., physical symptoms) [43].

### 4.2. Safety Climate in Relation to Perceptions of Safety, Mental Strain and Injury Event

In the present study, we identified that the dimensions of the safety climate were strongly related to personal perceptions of safety. The dimensions were also related to mental strain and, partly, to adverse events and injuries.

Injury events of individual team members tended to relate to perceived mental strain and to the two climate dimensions addressing teams’ shared perceptions of the team’s safety commitment, priority and non-risk acceptance. Though these correlations were fairly weak in this sample, these findings are reinforced by earlier research identifying positive relationships between safety climate and the safety-related behaviour and outcomes, in terms of, e.g., low injury rates [12]. That dimensions of peer safety commitment and non-risk acceptance seem to be of importance for adverse events and injuries is supported by narratives in the focus groups emphasising the team’s experience and agreements. It is plausible that the mediating role of peers [35] may have a more significant influence in jobs such as homecare with no, or only a few, peers close in time and space. This implies that such teams may develop strong safety practices despite management’s priorities. However, more important is their fragility in terms of conflict avoidance and the risk of developing bad practices’, as expressed by the participants in focus groups. In hierarchal organisations, homecare staff might not have the opportunity to influence the external prerequisites for their safe work performance. Earlier studies in homecare have reported a perceived lack of a supportive culture [16,44] and stressors such as, e.g., problems in interpersonal relationships and low decision latitude, are of significance for the risk of suffering injuries [45].

The Swedish work environment authority highlights injuries and mental strain as concerns in this sector. As work-related mental strain was negatively associated with safety climate dimensions, this measure may be an important indicator. A recent study showed that safety climate and psychosocial working conditions such as job control and support were related to health and safety practitioners’ health and mental well-being [46]. While mental strain impacts negatively on work performance [47], actual opportunities to influence the environment and perceived self-efficacy in managing situations despite barriers have emerged as important for work performance [26,46].

### 4.3. Methodological Issues

The sample is rather small and cross-sectional, and the survey data is analysed according to differences between 11 teams. An advantage of this mixed methods design was the option to run qualitative focus group for a deeper understanding of the topic. As such, the results reflect the voices of a representative group of homecare staff working in an urban settlement (population about 80,000) in northern Sweden. However, the sample is not representative of the country (where about 80% of the settlements are smaller) or of a general group of workers in this profession. Other possible biases could be recall bias, social expectations or protests. It is possible that other participants would have agreed to take part in the research if the recruitment had by-passed the managers. Issues of authority and dependencies may influence participants’ willingness to participate. Those who perceived the commitment to health and safety-promoting activities to be good might have been more inclined to participate. When studying social influences and intra-unit differences, in particular, it was notable that the response rate differed in the different work units. Furthermore, those who declined to participate were significantly younger than those who did participate, but they did not, however, differ in terms of the overall age range, sex or profession compared to the actual participants. In the analysis, we chose to exclude teams with few responders, to obtain a representative value of the teams’ shared perceptions of safety.

Potentially modifiable aspects of the work setting, areas in need of improvement, as well as good practices in some teams were identified. By using and building further on these results in future practice and research, positive changes can be brought about for all employees within the homecare services. To verify the results, a larger-scale investigation is needed. Inter-team differences can be analysed more deeply with an explanatory sequential mixed methods design, using survey results as a basis for topics in focus group discussions.

## 5. Conclusions

Significant diversity was identified between the 11 home care teams in five of seven areas of safety climate. The dimensions of safety climate were related to personal perceptions of safety and mental strain and, partly, to adverse events/injuries.

Important areas for improvement of safety and quality were: a need to define and agree on criteria for a safe working environment; to develop leadership prioritising safety at work; and management able to provide trust, support and time. A prerequisite for these agreements was improved authority and communication between all parties involved.

## Figures and Tables

**Table 1:** Respondent perceptions of the safety climate.

	Organisation (n = 133)	Team level (n = 11)		
Safety climate dimensions	Mean, SD	Mean, lowest & highest		*p* ^1^
1: Management safety priority and competence	3.1 ± 0.6	2.7	3.6	0.001
2: Management safety empowerment	3.1 ± 0.6	2.6	3.5	0.003
3: Management safety justice	3.4 ± 0.6	2.8	3.7	0.001
4: Workers’ safety commitment	3.2 ± 0.6	2.7	3.8	<0.001
5: Workers’ safety priority and non-risk acceptance	3.0 ± 0.6	2.6	3.5	0.001
6: Inter-peer safety communication, learning and competence	3.4 ± 0.5	3.1	3.7	0.114
7: Workers’ trust in the efficacy of safety systems	3.4 ± 0.5	3.2	3.7	0.688

^1^ Statistically significant differences between group means of 11 homecare work teams were analysed by one-way ANOVA. Significance level <0.05.

**Table 2:** The dimensions of safety climate in relation to personal perceptions of safety, mental strain and injury event.

	General Safety	Mental Strain	Injury Event
**Safety climate dimensions**			
1: Management safety priority and competence	**0.44**	**−0.31**	−0.07
2: Management safety empowerment	**0.51**	**−0.23**	−0.11
3: Management safety justice	**0.51**	**−0.29**	−0.05
4: Workers’ safety commitment	**0.50**	***−0.15***	**−0.18**
5: Workers’ safety priority and non-risk acceptance	**0.46**	**−0.20**	***−0.15***
6: Inter-peer safety communication, learning and competence	**0.50**	**−0.32**	−0.05
7: Workers’ trust in the efficacy of safety systems	**0.42**	**−0.20**	−0.09
**Personal perceptions**			
8: General safety		**−0.18**	−0.13
9: Mental strain			***0.16***
10: Injury event			

Pearson’s correlations (2-tailed) significant at the **0.01** and ***0.05*** level.
